# Overexpression of *TwSQS*, *TwSE*, and *TwOSC* Regulates Celastrol Accumulation in Cambial Meristematic Cells and Dedifferentiated Cells

**DOI:** 10.3389/fpls.2022.926715

**Published:** 2022-07-01

**Authors:** Yadi Song, Jiawei Zhou, Yifeng Zhang, Yujun Zhao, Xiujuan Wang, Tianyuan Hu, Yuru Tong, Luqi Huang, Wei Gao

**Affiliations:** ^1^Beijing Shijitan Hospital, Capital Medical University, Beijing, China; ^2^School of Traditional Chinese Medicine, Capital Medical University, Beijing, China; ^3^State Key Laboratory of Dao-di Herbs, National Resource Center for Chinese Materia Medica, China Academy of Chinese Medical Sciences, Beijing, China; ^4^School of Pharmaceutical Sciences, Capital Medical University, Beijing, China

**Keywords:** *Tripterygium wilfordii*, squalene synthase, squalene epoxidase, oxidosqualene cyclase, cambial meristematic cells

## Abstract

Squalene synthase (SQS), squalene epoxidase (SE), and oxidosqualene cyclase (OSC) are encoding enzymes in downstream biosynthetic pathway of triterpenoid in plants, but the relationship between three genes and celastrol accumulation in *Tripterygium wilfordii* still remains unknown. Gene transformation system in plant can be used for studying gene function rapidly. However, there is no report on the application of cambial meristematic cells (CMCs) and dedifferentiated cells (DDCs) in genetic transformation systems. Our aim was to study the effects of individual overexpression of *TwSQS*, *TwSE*, and *TwOSC* on terpenoid accumulation and biosynthetic pathway related gene expression through CMCs and DDCs systems. Overexpression vectors of *TwSQS*, *TwSE*, and *TwOSC* were constructed by Gateway technology and transferred into CMCs and DDCs by gene gun. After overexpression, the content of celastrol was significantly increased in CMCs compared with the control group. However, there was no significant increment of celastrol in DDCs. Meanwhile, the relative expression levels of *TwSQS*, *TwSE*, *TwOSC*, and terpenoid biosynthetic pathway related genes were detected. The relative expression levels of *TwSQS*, *TwSE*, and *TwOSC* were increased compared with the control group in both CMCs and DDCs, while the pathway-related genes displayed different expression trends. Therefore, it was verified in *T. wilfordii* CMCs that overexpression of *TwSQS*, *TwSE*, and *TwOSC* increased celastrol accumulation and had different effects on the expression of related genes in terpenoid biosynthetic pathway, laying a foundation for further elucidating the downstream biosynthetic pathway of celastrol through *T. wilfordii* CMCs system.

## Introduction

*Tripterygium wilfordii* Hook. f. (*T. wilfordii*) is a woody medicinal plant, and its dried root is a well-known traditional Chinese medicine widely used to treat immune dysregulation (Li et al., 2005), inflammation (Xu et al., 1985; Zhang W. et al., 2017), and tumor (Wang et al., 2016). The pharmacologically active constituents of *T. wilfordii* are terpenoids, mainly including diterpenoid and triterpenoid (Liu et al., 2011). Celastrol is a triterpenoid compound and considered to be one of the most likely natural products to develop into modern medicines (Corson and Crews, 2007), which can efficiently treat inflammatory diseases (Guo et al., 2017), tumor (Liu et al., 2019), obesity, and metabolic dysfunction (Ma et al., 2015).

In plants, terpenoids are biosynthesized by the mevalonate (MVA) pathway located in the cytoplasm and the 2-C-methyl-D-erythritol-4-phosphate (MEP) pathway located in the plastid (Vranova et al., 2013). The isopentenyl pyrophosphate (IPP) and its isomer dimethylallyl diphosphate (DMAPP), which are the common precursors of terpenoids, can transfer through the plastid envelope to link the MVA and MEP pathways (Rodriguez-Concepcion and Boronat, 2002). Farnesyl diphosphate synthase (FPS) converts one molecule of DMAPP and two molecules of IPP to form farnesyl pyrophosphate (FPP), and two molecules of FPP can be modified to squalene-by-squalene synthase (SQS), which is the first enzyme to catalyze the MVA pathway in the biosynthesis of the sterols and triterpenoids (Abe et al., 1994). Then, the rate-limiting enzyme squalene epoxidase (SE) (Han et al., 2010) converts squalene into 2,3-oxidosqualene (Zhou et al., 2018). Then, 2,3-oxidosqualene can be subsequently modified by oxidosqualene cyclase (OSC) to friedelin (Zhou et al., 2019), which will be finally transformed to celastrol through a series of biosynthetic reactions. So far, the downstream biosynthetic pathway of celastrol has not been clearly elucidated.

Recently, a stable gene transformation system of *T. wilfordii* suspension cells for studying gene function has been developed, which is fast, convenient, and efficient (Zhao et al., 2017). Cambial meristematic cells (CMCs) are innately undifferentiated cells derived from cambium in plants and they possess characteristics of plant stem cells (Ochoa-Villarreal et al., 2015). CMCs were commonly used as cell culture systems of medical plants, such as *Taxus cuspidata* (Lee et al., 2010), *Catharanthus roseus* (Moon et al., 2015), *Camptotheca acuminata* (Zhang Y. H. et al., 2017), and *Tripterygium wilfordii* (Song et al., 2019), from which CMCs could provide a cost-effective system for producing higher amounts of important natural products than typical dedifferentiated cells (DDCs). However, there is no report on the application of CMCs in genetic transformation system.

Overexpression of genes that encode enzymes in the biosynthetic pathway can directly affect gene expression levels and alter the yields of direct and end products associated with the encoding enzymes, which was extensively researched in plants. In adventitious roots of *Panax ginseng*, overexpressing *SQS* produced approximately two times more phytosterol than the wild type (Lee et al., 2004). After *SE* overexpression, the content of ganoderic acid in *Ganoderma lingzhi* was two times higher than that of the wild type (Zhang D. H. et al., 2017). In *Populus davidiana*, overexpression of *OSC* increased friedelin accumulation by 233–445% compared with the non-transgenic plants (Han et al., 2019). However, the effects of overexpression of *TwSQS*, *TwSE*, and *TwOSC* on celastrol biosynthesis in *T. wilfordii* CMCs and DDCs still remain unknown.

In this study, we aimed to analyze the effects of individual overexpression of *TwSQS*, *TwSE*, and *TwOSC* on celastrol accumulation and terpenoid biosynthetic pathway related gene expression through *T. wilfordii* CMCs and DDCs, laying a foundation for further research on downstream biosynthetic pathway of celastrol.

## Materials and Methods

### Plant Cell Suspension Cultures of *Tripterygium wilfordii* Cambial Meristematic Cells and Dedifferentiated Cells

The plant *Tripterygium wilfordii* Hook. f. was obtained from Yongan national forest in Fujian Province, China. CMCs and DDCs were isolated from the stem of *T. wilfordii* plant and cultured in Murashige and Skoog (MS) medium (PhytoTechnology Laboratories, Lenexa, KS, United States) as described previously (Song et al., 2019). Briefly, under the sterile condition, cambium, phloem, cortex, and epidermal tissues of the *T. wilfordii* stem were peeled off from the xylem. The epidermal tissue side was laid on MS solid medium that contained 2 mg/L 2,4-dichlorophenoxyacetic acid (2,4-D), 2 mg/L naphthalene-acetic acid (NAA), 30 g/L sucrose, and 8 g/L agar. The pH value of medium was 5.8–6.0. During the culture process, CMCs were formed from the cambium, and DDCs were formed from the phloem, cortex, and epidermis. The characteristic features of CMCs and DDCs were previously identified by our team (Song et al., 2019). Notably, 2.5 g (fresh cell weight) cells from each cell line were cultured in 100 ml Erlenmeyer flasks including 40 ml of MS liquid medium. The composition of the liquid medium is the same as the solid medium except for agar. The flasks were placed at 25°C in dark and agitated at 120 rpm. Subculturing was undertaken at 20-day intervals.

### Construction of Entry Vectors

The full-length cDNA of *TwSQS* (Liu et al., 2016), *TwSE* (Zhou et al., 2018), and *TwOSC* (Zhou et al., 2019) (GenBank accession numbers: *TwSQS* KR401220, *TwSE* MG717396, and *TwOSC* KY885467) was previously cloned. The full open reading frames (ORF) of *TwSQS*, *TwSE*, and *TwOSC* were amplified by PCR using specific overexpression (OE) primers (Supplementary Table 1) and Phusion High-Fidelity Master Mix (New England Biolabs, United States). Their plasmids were used as templates. After detecting that the sequence lengths of the amplified products were the same as their full ORF, the products were purified and then inserted into pENTR^TM/^D-TOPO entry vectors using the Cloning Kit (Thermo Fisher Scientific, United States). The recombinant vectors were transformed into *Escherichia coli* (*E. coli*). Trans5α cells (TransGen Biotech, Beijing, China) and then cultured on Luria-Bertani (LB) solid medium with 50 mg/L kanamycin for selection of entry vectors. M13 F/R primers (Supplementary Table 1) were used to verify that entry vectors were successfully constructed.

### Construction of Overexpression Vectors

The overexpressed target fragments of *TwSQS*, *TwSE*, and *TwOSC* were transferred from entry vectors into pH7WG2D vectors using the Gateway LR Clonase TM II Enzyme Mix (Thermo Fisher Scientific), respectively. The promoter of the vector was p35S and the terminator was T35S. The recombinant vectors containing each fragment were transformed into *E. coli* Trans5α cells individually and selected on LB solid medium with 50 mg/L spectinomycin, followed by pH7 (Supplementary Table 1) sequencing. OE primers (Supplementary Table 1) were used to verify that overexpression vectors were successfully constructed. The plasmids of *TwSQS*, *TwSE*, and *TwOSC* overexpression vectors were extracted using EZNA^®^ Plasmid Maxi Kit (OMEGA, Norcross, GA, United States).

### Transfer of Overexpression Vectors Into *Tripterygium wilfordii* Cambial Meristematic Cells and Dedifferentiated Cells

At the time of subculturing, CMCs and DDCs were cultured on MS solid medium containing 2 mg/L 2,4-D, 2 mg/L NAA, 30 g/L sucrose, and 8 g/L agar. The pH value of medium was 5.8–6.0. Notably, 200 mg (fresh cell weight) cells corresponded to 3 ml medium. Cells grew in dark at 25°C for 7 days before transfer. The plasmids of overexpression vectors were separately transferred into *T. wilfordii* CMCs and DDCs using Gene gun instrument (PDS 100/He, Bio-Rad), and the Gene gun protocol has been described previously in detail (Zhao et al., 2017). Briefly, the plasmids were mixed with 1 μm gold microparticles (Bio-Rad), and the mixture was then bombarded into the cells using Gene gun under high-pressure helium. The empty vector pH7WG2D was transferred, respectively, into CMCs and DDCs as a control check. Then, the cells were cultured in the original medium in dark at 25°C for 7 days. After 7 days, the cells were separated from the culture medium, and the cells were stored at −80°C for later use. All samples had five biological replicates.

### Verification of Successful Transfer of Overexpression Vectors in Cells

The cell samples were divided into two parts, i.e., one for terpenoids content measurement and the other for gene expression determination. Total RNAs from all cell samples were extracted using the Total RNA Extraction Kit (Promega, Shanghai, China). Then, all RNAs were reverse-transcribed to first-strand cDNAs using the FastQuant RT kit (with gDNase) (Tiangen Biotech, Beijing, China). The hygromycin (Hyg) fragments were amplified in the cDNAs using Hyg F/R primers (Supplementary Table 1) to verify the successful transfer of overexpression vectors in *T. wilfordii* CMCs and DDCs.

### Extraction and Determination of Terpenoids in *Tripterygium wilfordii* Cambial Meristematic Cells and Dedifferentiated Cells

The remaining cell samples were freeze-dried *in vacuo* for 48 h. Then, 5 mg dried cells were precisely weighed and soaked in 250 μl n-hexane for overnight at 4°C. Another 5 mg dried cells were also precisely weighed and soaked in 250 μl 80% (v/v) methanol for overnight at 4°C. All cells were ultrasonicated at 25°C and 40 kHz for 1 h the next day. After centrifugation at 10,000 × *g* for 10 min, the supernatants were filtered through 0.22 μm PTFE microporous membrane. For determining the content of squalene (direct product of TwSQS enzyme), 2,3-oxidosqualene (direct product of TwSE enzyme), and friedelin (direct product of TwOSC enzyme), the n-hexane extracts were detected using GC-MS (Agilent 7000, Agilent) with a DB-5ms capillary column (15 m × 250 μm × 0.1 μm) as described previously (Zhou et al., 2019). The gas chromatography detection conditions were set as follows: the initial column temperature was 50°C, hold for 1 min, increased to 260°C at a rate of 50°C/min, further increased to 272°C at a rate of 1°C/min, and hold for 4 min. The injection temperature was 250°C and the split ratio was 20: 1. The carrier gas was helium, and the flow rate was 1 ml/min. The mass spectrometric detector was operated with ionization energy of 70 eV by electron impact. The ion trap temperature was 230°C, and the spectra were recorded in the range of 10–550 m/z. The sample injection volume was 1 μl.

For determining the content of celastrol, the methanol extracts were detected using UPLC (1290 Infinity II, Agilent) with an ACQUITY UPLC^®^ HSS T3 chromatographic column (1.8 μm, 2.1 mm × 100 mm, Waters). Column temperature was 40°C and the flow rate was 0.4 ml/min. Gradient elution with 100% acetonitrile (mobile phase A) and 0.1% (*v*/*v*) formic acid in water (mobile phase B) was used: 0 min: 70% B, 5 min: 65% B, 15 min: 30% B, 21 min: 10% B. The UV detector was monitored at 426.0 and 220 nm. The sample injection volume was 5 μl.

### Relative Expression Analysis of *TwSQS*, *TwSE*, and *TwOSC*

The determination of gene expression levels was carried out by quantitative real-time reverse transcription-polymerase chain reaction (qRT-PCR). KAPA SYBR^®^ FAST qPCR Master Mix Kit (KAPA Biosystems, Wilmington, MA, United States) was used to perform qRT-PCR experiment on the QuantStudio 5 real-time PCR system (Thermo Fisher Scientific). The cDNA obtained above was used as the templates for qRT-PCR. The qRT-PCR mixture contained 10 μl of 2 × qPCR Master Mix, 1 μl of cDNA (10 ng), 0.4 μl of 50 × ROX reference Dye low, 0.4 μl of primers (10 μM), and 8.2 μl of double distilled H_2_O. The qRT-PCR experiment was performed under the following conditions: at 95°C for 3 min, followed by 40 cycles consisted of 95°C for 3 s and 60°C for 30 s, and then a melting curve cycle: 95°C for 1 s and 60°C for 20 s. *Elongation factor 1*α (*Ef1*α) was used as the reference gene, and the relative expression was analyzed by the 2[-Delta Delta C(T)] method (Livak and Schmittgen, 2001) with five biological replicates and three technical replicates. The primer sequences employed are listed in Supplementary Table 1.

### Relative Expression Analysis of Genes Involved in Terpenoid Biosynthetic Pathway

We also analyzed the expression levels of genes encoding *T*. *wilfordii* 1-deoxy-D-xylulose-5-phosphate synthase (TwDXS), geranylgeranyl diphosphate synthase (TwGGPPS), hydroxymethylglutaryl-CoA synthase (TwHMGS), hydroxymethylglutaryl-CoA reductase (TwHMGR), isopentenyl diphosphate isomerase (TwIDI), TwFPS, TwSQS, TwSE, and TwOSC enzymes under the conditions of *TwSQS*, *TwSE*, and *TwOSC* overexpression. These enzymes were involved in biosynthetic pathway of terpenoids in *T. wilfordii*. The specific qRT-PCR primers used are listed in Supplementary Table 1. The methods of relative gene expression analysis were same as described in the relative expression analysis of *TwSQS*, *TwSE*, and *TwOSC*.

## Results

### Confirmation of *TwSQS*, *TwSE*, and *TwOSC* Overexpression Vectors

**FIGURE 1:**
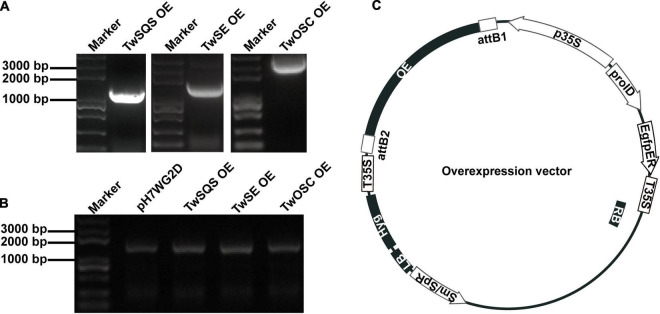
Construction and transfer verification of *TwSQS*, *TwSE*, and *TwOSC* overexpression vectors. **(A)** *TwSQS*, *TwSE*, and *TwOSC* overexpression fragments. *TwSQS* overexpression fragment of 1,241 bp, *TwSE* overexpression fragment of 1,584 bp, and *TwOSC* overexpression fragment of 2,298 bp. **(B)** Verification of successful transfer of overexpression vectors in *Tripterygium wilfordii* CMCs and DDCs. Agarose gel electrophoresis showed the hygromycin fragment (1,787 bp). **(C)** Schematic diagram of overexpression vectors. OE, overexpression; Hyg, hygromycin.

**FIGURE 2:**
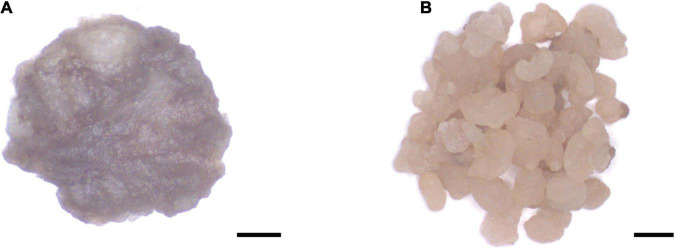
The morphology of *Tripterygium wilfordii* CMCs and DDCs. **(A)** CMCs were mostly aggregated by small cell groups. Scale bar, 2 mm. **(B)** DDCs were gathered with large cell groups. Scale bar, 2 mm.

The *TwSQS* overexpression fragment of 1,241 bp, the *TwSE* overexpression fragment of 1,584 bp, and the *TwOSC* overexpression fragment of 2,298 bp were obtained. The sequencing results were consistent with the ORF sequence of *TwSQS*, *TwSE*, and *TwOSC*, confirming that overexpression vectors of the three genes were successfully constructed. Agarose gel electrophoresis showed the length of the target fragments in Figure 1A. The cDNAs of CMCs and DDCs after transfer were amplified by specific primers and the Hyg fragments with the length of 1,787 bp were found in all cell samples (Figure 1B), indicating that the empty vector pH7WG2D and overexpression vectors were successfully transferred into CMCs and DDCs. A schematic diagram of overexpression vector is shown in Figure 1C, and the morphology of CMCs and DDCs is shown in Figure 2. The texture of CMCs was softer than that of DDCs.

### Accumulation of Squalene, 2,3-Oxidosqualene, and Friedelin in *Tripterygium wilfordii* Cambial Meristematic Cells and Dedifferentiated Cells

**FIGURE 3:**
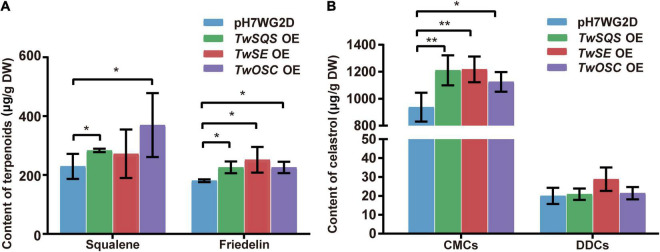
Accumulation of squalene, friedelin, and celastrol in *Tripterygium wilfordii* CMCs and DDCs after overexpressing *TwSQS*, *TwSE*, and *TwOSC.* **(A)** Accumulation of squalene and friedelin in CMCs. **(B)** Accumulation of celastrol in CMCs and DDCs. OE, overexpression; DW, dry weight. The data are summarized as means ± standard deviation. *p < 0.05 and **p < 0.01, *n* = 5. Student’s *t*-test.

To explore the ability of TwSQS, TwSE, and TwOSC enzymes to biosynthesize related terpenoids after overexpression, we determined the content of direct products of the three enzymes in *T. wilfordii* CMCs and DDCs (Figure 3A; Supplementary Figures 1–3). The contents of squalene and friedelin were detected in CMCs, but not in DDCs. In the pH7WG2D control group, *TwSQS* overexpression group, and *TwOSC* overexpression group, the content of squalene was 229.5 ± 42.4, 283.5 ± 5.6, and 369.8 ± 108.7 μg/g, respectively. The content of friedelin was 180.8 ± 4.7, 226.2 ± 20.2, 251.6 ± 43.7, and 225.9 ± 19.4 μg/g in the pH7WG2D control group, *TwSQS* overexpression group, *TwSE* overexpression group, and *TwOSC* overexpression group, respectively. Only the content of squalene determined in the *TwSE* overexpression group had no significant differences compared with the control group. However, we did not detect any 2,3-oxidosqualene in both CMCs and DDCs.

### Accumulation of Celastrol in *Tripterygium wilfordii* Cambial Meristematic Cells and Dedifferentiated Cells

The content of celastrol was determined in *T. wilfordii* CMCs and DDCs after overexpression (Figure 3B; Supplementary Figure 4). Compared with the control group, the content of celastrol in CMCs overexpression group showed an increase with statistical difference. In the pH7WG2D control group, *TwSQS* overexpression group, *TwSE* overexpression group, and *TwOSC* overexpression group, the content of celastrol in CMCs was 937.6 ± 106.9, 1,209.8 ± 111.3, 1,217.6 ± 95.0, and 1,125.2 ± 95.7 μg/g, respectively. In contrast to this, there was no significant difference in the content of celastrol between DDCs overexpression group and the control group.

### Relative Expression Analysis of *TwSQS*, *TwSE*, and *TwOSC* in *Tripterygium wilfordii* Cambial Meristematic Cells and Dedifferentiated Cells

**FIGURE 4:**
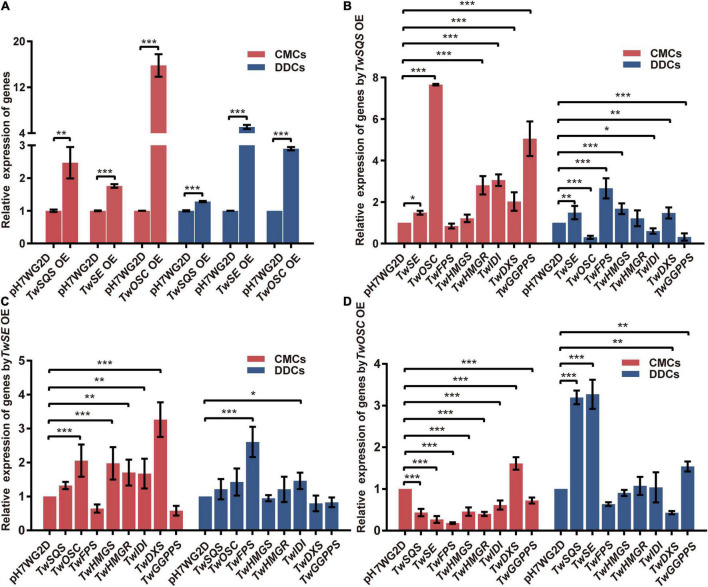
Relative expression analysis of related genes involved in terpenoid biosynthetic pathway in *Tripterygium wilfordii* CMCs and DDCs after overexpression of *TwSQS*, *TwSE*, and *TwOSC*. **(A)** Relative expression levels of *TwSQS*, *TwSE*, and *TwOSC.* **(B)** Relative expression levels of related genes in terpenoid biosynthetic pathway after *TwSQS* overexpression. **(C)** Relative expression levels of related genes in terpenoid biosynthetic pathway after *TwSE* overexpression. **(D)** Relative expression levels of related genes in terpenoid biosynthetic pathway after *TwOSC* overexpression. OE, overexpression. The data are summarized as means ± standard deviation. *p < 0.05, **p < 0.01, and ***p < 0.001, *n* = 5. Student’s *t*-test.

To further investigate the transcription levels of overexpressed genes, the relative expression levels of *TwSQS*, *TwSE*, and *TwOSC* were determined by qRT-PCR (Figure 4A). In CMCs, relative expression levels of *TwSQS*, *TwSE*, and *TwOSC* increased by 2.5, 1.8, and 15.8 times compared with the pH7WG2D control group, respectively. As for DDCs, their relative expression levels exhibited 1. 3-, 5. 1-, and 2.9-fold increase compared with the control group, respectively. In both cell lines, the relative expression levels of the three genes showed statistical significance compared with the control group.

### Relative Expression Analysis of Other Important Genes Involved in Terpenoid Biosynthetic Pathway

Next, we analyzed the relative expression levels of other genes encoding important enzymes involved in terpenoid biosynthetic pathway after overexpression of *TwSQS*, *TwSE*, and *TwOSC*, respectively.

Under the *TwSQS* overexpression condition (Figure 4B), in terms of CMCs group, the relative expression levels of *TwSE*, *TwOSC*, *TwHMGR*, *TwIDI*, *TwDXS*, and *TwGGPPS* increased by 1.5, 7.7, 2.8, 3.1, 2.0, and 5.0 times, respectively, compared with the pH7WG2D control groups. The relative expression levels of *TwFPS* and *TwHMGS* were not statistically significant compared with the control group. As for DDCs group, in contrast to the relative expression levels of *TwOSC*, *TwIDI*, and *TwGGPPS* decreasing to 0. 3-, 0. 6-, and 0.3-fold, respectively, the relative expression levels of *TwSE*, *TwFPS*, *TwHMGS*, and *TwDXS* increased by 1.5, 2.7, 1.7, and 1.5 times, respectively, compared with the control group. Only the relative expression level of *TwHMGR* was not statistically significant.

Under the *TwSE* overexpression condition (Figure 4C), in terms of CMCs group, the relative expression levels of *TwOSC*, *TwHMGS*, *TwHMGR*, *TwIDI*, and *TwDXS* increased by 2.1, 2.0, 1.7, 1.7, and 3.3 times, respectively, compared with the control group. The relative expression levels of *TwSQS*, *TwFPS*, and *TwGGPPS* were not statistically significant. As for the DDCs group, only the relative expression levels of *TwFPS* and *TwIDI* were statistically significant, increasing by 2.6 and 1.5 times compared with the control group, respectively.

Under the *TwOSC* overexpression condition (Figure 4D), in terms of CMCs group, apart from an increase in relative expression level of *TwDXS* by 1.61 times, the relative expression levels of *TwSQS*, *TwSE*, *TwFPS*, *TwHMGS*, *TwHMGR*, *TwIDI*, and *TwGGPPS* reduced by 0. 4-, 0. 3-, 0. 2-, 0. 5-, 0. 4-, 0. 6-, and 0.7-fold compared with the control group, respectively. The relative expression levels of all genes determined were statistically significant. As for the DDCs group, compared with the control group, the relative expression levels of *TwSQS*, *TwSE*, and *TwGGPPS* increased by 3.2, 3.3, and 1.5 times, respectively, but the relative expression level of *TwDXS* decreased to 0.4-fold. The expression levels of *TwFPS*, *TwHMGS*, *TwHMGR*, and *TwIDI* were not statistically significant.

## Discussion

In our study, we obtained the overexpression vectors of *TwSQS*, *TwSE*, and *TwOSC*, and they were transferred into *T. wilfordii* CMCs and DDCs individually. After overexpression of each gene, the content of triterpenoid celastrol increased in CMCs, but not in DDCs. Meanwhile, the relative expression levels of *TwSQS*, *TwSE*, and *TwOSC* were all enhanced in CMCs. These results indicate that *TwSQS*, *TwSE*, and *TwOSC* were involved in the biosynthesis of celastrol. Furthermore, overexpression of *TwSQS*, *TwSE*, and *TwOSC* had a stronger effect on the biosynthesis of celastrol in CMCs than in DDCs.

In this study, the direct product 2,3-oxidosqualene of TwSE enzyme was not detected in both *T. wilfordii* CMCs and DDCs after overexpression of *TwSE*. The reason is probably that 2,3-oxidosqualene contains an epoxy group in the molecule, which is usually unstable and may be easily degraded or converted into other compounds (Araki et al., 2016). Another possibility could be that in other biosynthetic pathways, such as sterol and non-steroidal triterpene biosynthetic pathways, the immediate utilization of the substrate 2,3-oxidosqualene by other downstream enzymes is highly efficient, such as lanosterol synthase and cycloartenol synthase (Babiychuk et al., 2008). Besides, in this study, squalene and friedelin were detected in CMCs, but not in DDCs. The reason for this phenomenon might be that intermediate products in DDCs were likely to transform into other compounds (such as sterol) soon before detection. Furthermore, overexpression of a gene involved in terpenoid biosynthetic pathway might lead to increase the flux toward the steroid biosynthesis pathway (Engels et al., 2008), which could be the reason why no significant increase in celastrol was detected in DDCs. The biosynthetic pathways of *T. wilfordii* terpenoids and steroids are so complex that further research is required to fully elucidate them.

**FIGURE 5:**
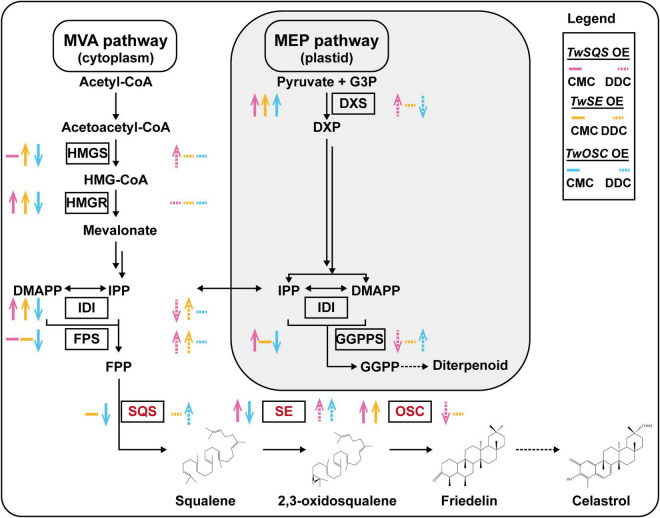
The expression trends of genes involved in terpenoid biosynthetic pathway after overexpression of *TwSQS*, *TwSE*, and *TwOSC* based on Figure 4. A black single arrow represents a one-step reaction, a black double arrow represents a multistep reaction, and a black dotted arrow represents unknown steps. The colored upward or downward arrows indicate corresponding increase and decrease in gene expression. Colored horizontal line represents no statistical significance between the overexpression group and the pH7WG2D control group. The SQS, SE, and OSC enzymes analyzed in this study are highlighted in red. OE, overexpression.

Overexpression of terpenoid biosynthetic enzyme genes may affect the expression of other genes in the pathway (Tong et al., 2019; Zhang et al., 2020). In this study, the relative expression levels of terpenoid biosynthetic pathway related genes showed different trends after overexpression of *TwSQS*, *TwSE*, and *TwOSC* in *T. wilfordii* CMCs and DDCs (Figure 5), resulting in different effects on the biosynthesis of terpenoids. In addition, after overexpression of *TwSQS*, *TwSE*, and *TwOSC* in CMCs, the relative expression of *TwDXS* pathway was increased, leading to an increase in the content of celastrol, which indicates that *TwDXS*, *TwSQS*, *TwSE*, and *TwOSC* seem to have synergistic effects during the biosynthesis of celastrol. Moreover, after *TwOSC* overexpression, though the relative expression levels of terpenoid biosynthetic pathway related genes detected in CMCs were all decreased except *TwDXS*, the content of celastrol was still increased, which could be attributed to the feedback regulation on the expression of genes in the pathway (Zhang et al., 2018).

In this study, the content of celastrol increased in CMCs, but there was no significant increment of celastrol in DDCs after overexpression of each gene. This may be due to the morphological difference between the two cell lines. Studies have shown that vacuoles in plant cells can dynamically store secondary metabolites, including terpenoids, alkaloids, and flavonoids (De Brito Francisco and Martinoia, 2018). In a previous study, we found that the morphology of CMCs and DDCs was quite different. A single CMC had a number of vacuoles, while a single DDC only had one vacuole (Song et al., 2019). Compared with one vacuole in the DDC, the large number of vacuoles could allow celastrol to be stored in the CMC as much as possible before detection. Moreover, the content of celastrol produced in CMCs was 57. 9-, 37. 5-, and 53.3-fold higher than that of DDCs after *TwSQS*, *TwSE*, and *TwOSC* overexpression, respectively. In addition to the morphological difference of two cell lines, differences in gene expression may contribute to this result. The relative expression levels of *TwSQS* and *TwOSC* in CMCs were higher than that of DDCs after overexpression. Higher gene expression might contribute to higher celastrol accumulation in CMCs. Our results demonstrate that CMCs could be used as a better system than DDCs to study the function of genes involved in the biosynthetic pathway of celastrol.

After gene overexpression, the content of celastrol produced by the empty vector of CMCs was 46.9 times higher than that of DDCs. It is probably due to the different degrees of stimulation caused by the same empty vector in two different cell lines. As depicted from the results of Figures 3B, 4A, among the three genes, in CMCs, although *TwSE* had the lowest expression level after overexpression alone, it has the most increased celastrol content. While the expression level of *TwOSC* was highest after overexpression alone, the increase in celastrol content was minimal. After overexpression alone, the expression level of *TwOSC* and the increase in celastrol content were moderate. In DDCs, although expression levels of the three genes increased after overexpression, the celastrol content did not increase with a significant difference. As mentioned above, overexpression of *TwSE* was more conducive to the production of celastrol, while overexpression of *TwSQS* had less effect on that among the three genes.

In terms of the effects of downstream genes (Figures 4B–D), when *TwSQS* was overexpressed alone, in CMCs, the expression levels of *TwSE* and *TwOSC* increased; In DDCs, the expression level of *TwSE* increased, but the expression level of *TwOSC* decreased. When *TwSE* was overexpressed alone, in CMCs, the expression levels of *TwSQS* and *TwOSC* increased, but there was no significant difference in *TwSQS*; In DDCs, the expression levels of *TwSQS* and *TwOSC* increased without a significant difference. When *TwOSC* was overexpressed alone, the expression levels of *TwSQS* and *TwSE* decreased in CMCs and increased in DDCs. Moreover, SE is a rate-limiting enzyme in the biosynthetic pathway of terpenoids (Han et al., 2010). In the future, we can further explore the effect of *TwSE* on celastrol biosynthesis in CMCs to fully clarify how *TwSE* increases celastrol content in terms of molecular mechanism. We can also utilize the CMCs system to extract other functional unknown genes in the downstream biosynthetic pathway of celastrol.

In this study, it was the first time that CMCs had been applied to a genetic transformation system. Compared with the stable gene transformation system of suspension cells, CMCs system exhibited unique enchantment. Most CMCs present as a single cell or as clusters of small cells, whereas suspension cells are present as large aggregates (Song et al., 2019). Less aggregation may not form non-productive microenvironments and increase the nutrient and oxygen supply to CMCs (Ochoa-Villarreal et al., 2015), resulting in a more stable growth state of CMCs. Besides, shear stress is a crucial limitation in the scale-up of plant cells, affecting cells by causing death due to fluid motion (Joshi et al., 1996). The reduced aggregation size of CMC decreases sensitivity of shear stress. The characteristics of numerous vacuoles and thin cell wall in CMCs are also thought to increase tolerance to shear stress (Lee et al., 2010; Song et al., 2019). When cultured in a 20-L air-lift bioreactor, *Taxus cuspidata* (*T*. *cuspidata*). CMCs grew strikingly faster and healthier compared with DDCs, which turned into necrotic cells largely and rapidly (Lee et al., 2010). The production of paclitaxel in *T*. *cuspidata* CMCs was considerably higher than that of DDCs. Furthermore, an important factor that negatively impacts production of natural products is the heterogeneous nature of cells. Suspension cells consist of a mixture of different cell types, resulting in a high level of heterogeneity (Lee et al., 2010). As CMCs are innately undifferentiated cells with plant stem cell-like property, CMCs bypass the negative effects of the differentiation step, offering stability in product accumulation. CMCs are also highly responsive to elicitation (Lee et al., 2010; Song et al., 2019). The utilization of CMCs system provides a promising platform for the production of natural products.

Hairy root system can offer high production of natural products. Hairy roots from cocultivation accumulated 1,086 μg/g (fresh weight) of encecalin, which is more than three times higher than that of suspension cells system (Hernández-Altamirano et al., 2020). Hairy root system has also gained attention for the production of pharmacologically functional proteins (Parsons et al., 2010). But there is no report on CMCs in these fields. However, an important limitation in the production of natural products from hairy roots is that the target molecule must be biosynthesized within the roots of the original plant. This is significant because relatively few natural products are synthesized in root tissues, with the majority produced in aerial parts (Li and Wang, 2021). Whereas CMCs are obtained from cambium tissue in plants (Lee et al., 2010), they are not limited to the roots of plants. Moreover, a major limitation for the industrial-scale production of natural products using hairy roots is the development of appropriate bioreactors. The complex fibrous structure of the roots makes it difficult for a large-scale culture system. Hairy root growth is not homogeneous, which affects the reactor performance (Wilson et al., 1987), but CMCs are not subjected to the limitations mentioned above, and they could grow stably in 3-ton bioreactor with high performance (Lee et al., 2010). However, there is no report on the comparison of CMCs and hairy root in genetic transformation and accumulation of natural products. These require further studies to comprehensively compare the differences between CMCs and other systems.

In conclusion, it was verified in *T. wilfordii* CMCs that overexpression of *TwSQS*, *TwSE*, and *TwOSC* increased celastrol accumulation and had different effects on the expression of related genes in terpenoid biosynthetic pathway, which laid a foundation for further elucidating the downstream biosynthetic pathway of celastrol through *T. wilfordii* CMCs system. The established CMC system provides a useful tool to analyze other functional unknown genes in *T. wilfordii* in the near future.

## Data Availability Statement

The datasets presented in this study can be found in online repositories. The names of the repository/repositories and accession number(s) can be found in the article/Supplementary Material.

## Author Contributions

YS, XW, and WG conceived and designed the research. YS conducted the experiments and wrote the manuscript. JZ assisted the vector construction. YFZ and YJZ assisted the gene gun technology. TH, YT, and LH assisted the data analysis. All authors had given approval to the final version of the manuscript.

## Conflict of Interest

The authors declare that the research was conducted in the absence of any commercial or financial relationships that could be construed as a potential conflict of interest.

## Publisher’s Note

All claims expressed in this article are solely those of the authors and do not necessarily represent those of their affiliated organizations, or those of the publisher, the editors and the reviewers. Any product that may be evaluated in this article, or claim that may be made by its manufacturer, is not guaranteed or endorsed by the publisher.
