# Supplementary material for: Overexpression of TwSQS, TwSE, and TwOSC Regulates Celastrol Accumulation in Cambial Meristematic Cells and Dedifferentiated Cells
Source: Front Plant Sci. 2022 Jul 1;13:926715. doi: 10.3389/fpls.2022.926715 (PMC9284119; doi:10.3389/fpls.2022.926715)
Supplement: Supplementary file 1 [file Data_Sheet_1.pdf]

Supporting information

Overexpression of *TwSQS*, *TwSE* and *TwOSC* regulates celastrol accumulation in cambial meristematic cells and dedifferentiated cells

Yadi Song[1,2], Jiawei Zhou[2], Yifeng Zhang[1], Yujun Zhao[3], Xiujuan Wang[2, *], Tianyuan Hu[2], Yuru Tong[4], Luqi Huang[3], Wei Gao[1, 2, *]

[1] Beijing Shijitan Hospital, Capital Medical University, Beijing, 100038, China

[2] School of Traditional Chinese Medicine, Capital Medical University, Beijing, 100069, China

[3] State Key Laboratory of Dao-di Herbs, National Resource Center for Chinese Materia Medica, China Academy of Chinese Medical Sciences, Beijing, 100700, China

[4] School of Pharmaceutical Sciences, Capital Medical University, Beijing, 100069, China

*Corresponding authors

Xiujuan Wang: Tel: 86-10-83911671, Fax: 86-10-83911627, E-mail: wxj0517@sina.com

Wei Gao: Tel: 86-10-83916572, Fax: 86-10-83911627, E-mail: weigao@ccmu.edu.cn

A list of the contents

Figure S1 GC-MS analysis of squalene and friedelin standards and samples in CMCs of *T. wilfordii*

Figure S2 GC-MS analysis of squalene and friedelin standards and samples in DDCs of *T. wilfordii*, indicating that there was no squalene and friedelin in DDCs

Figure S3 Mass spectrometry of squalene and friedelin standards and samples in CMCs of *T. wilfordii*.

Figure S4 UPLC chromatograms of celastrol standard and samples.

Table S1 Primers used in this study

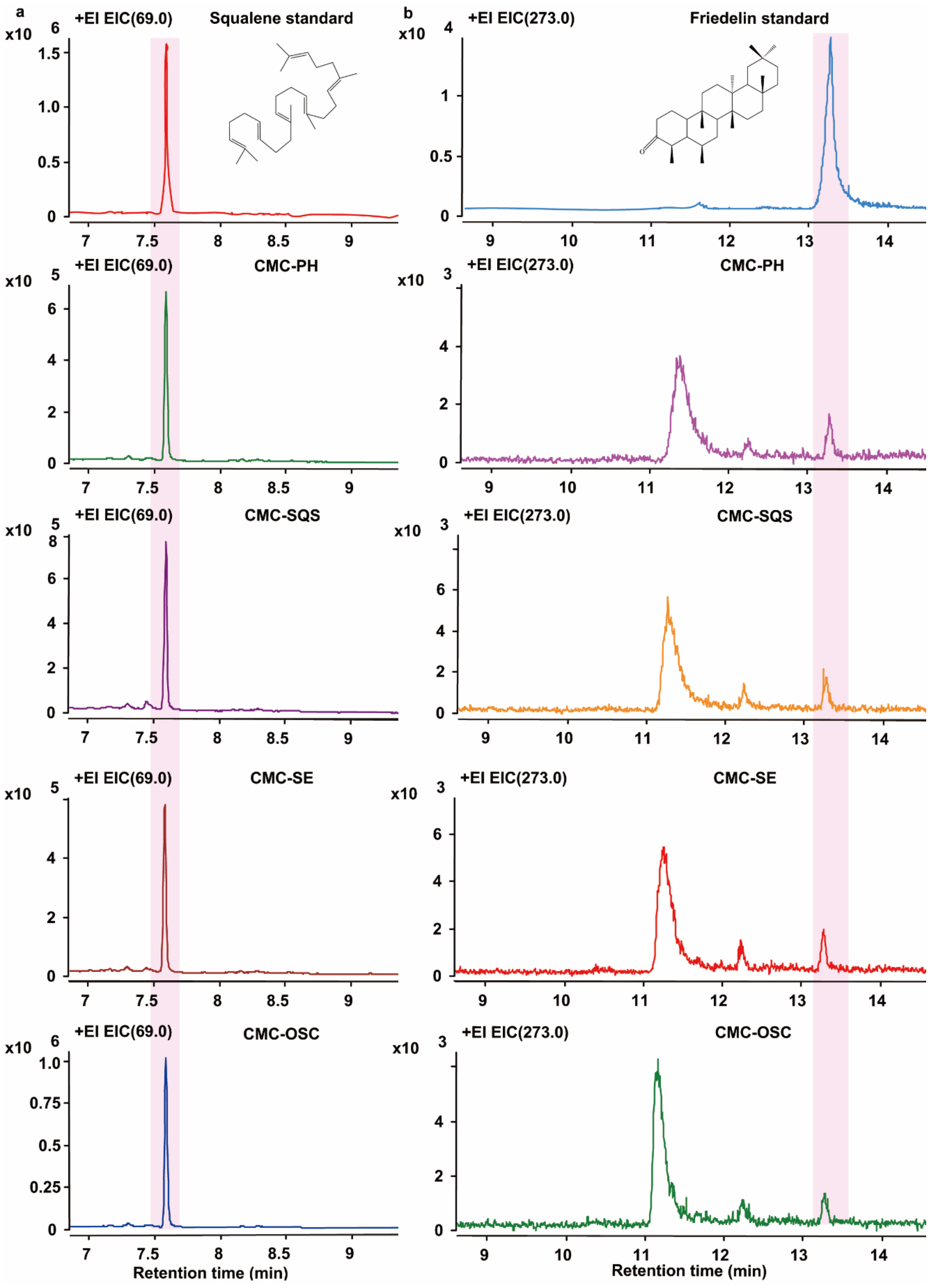

Figure S1 GC-MS analysis of squalene **a** and friedelin **b** standards and samples in CMCs of *T. wilfordii*.

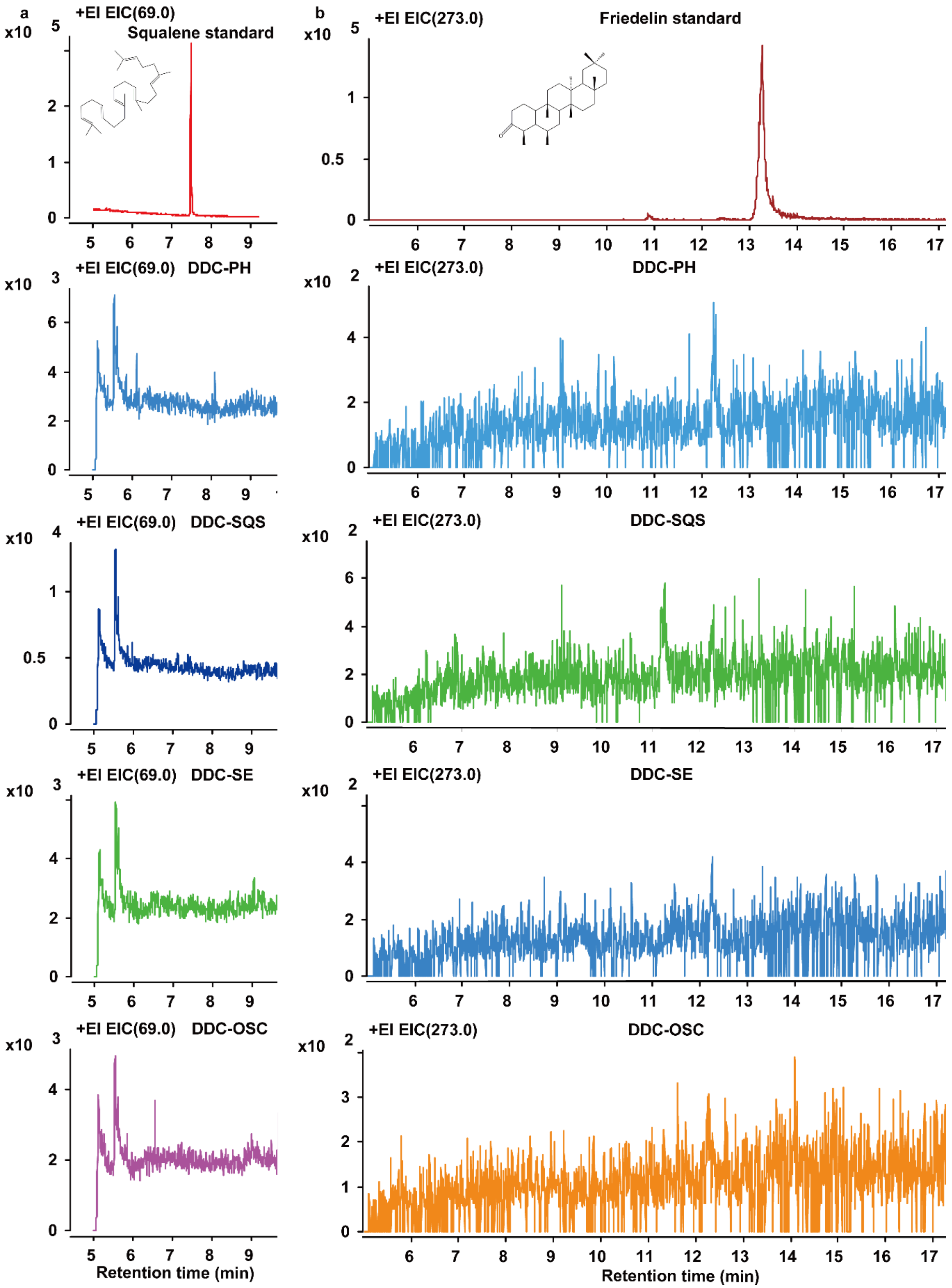

Figure S2 GC-MS analysis of squalene. **a** and friedelin **b** standards and samples in DDCs of *T. wilfordii*.

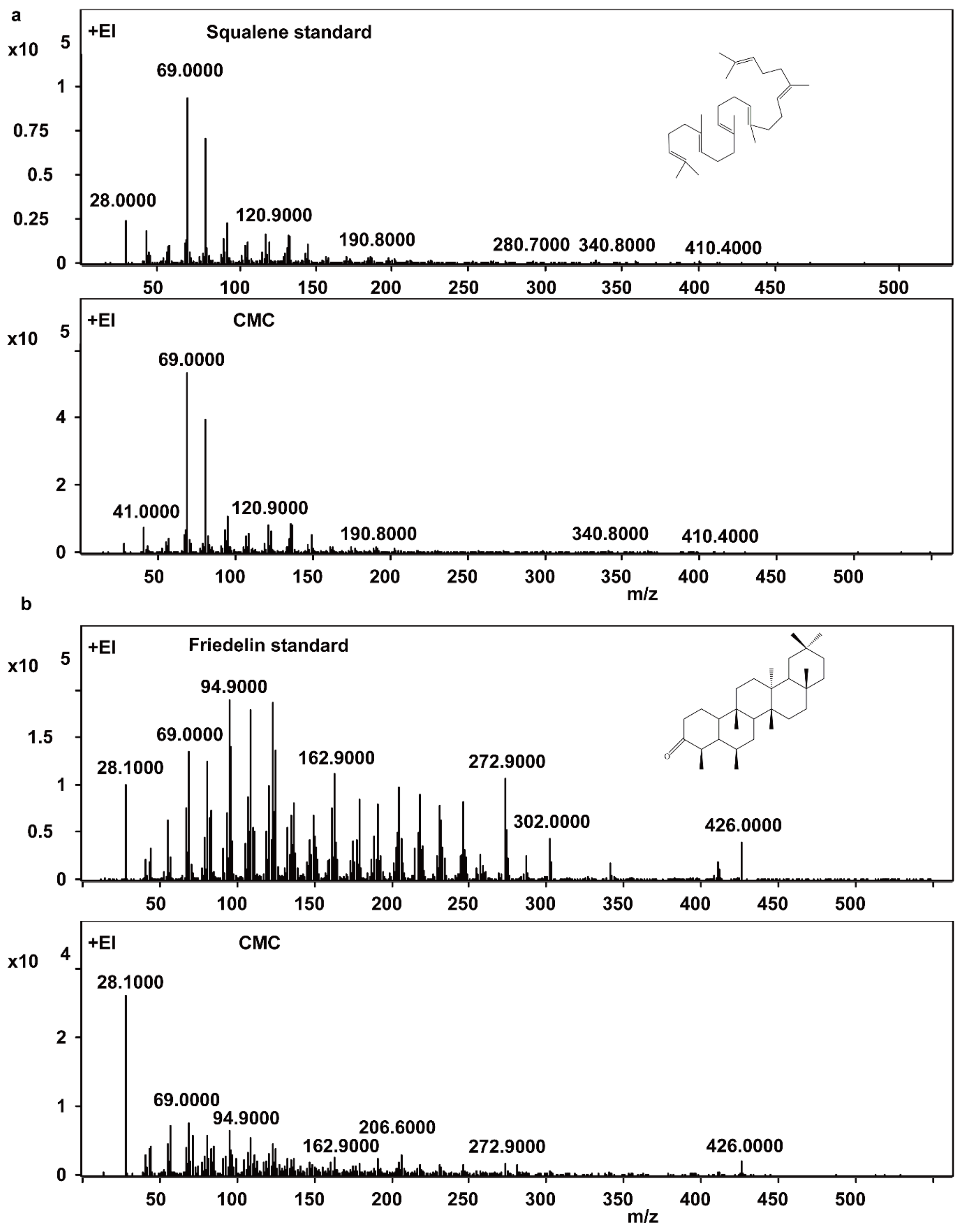

Figure S3 Mass spectrometry of squalene **a** and friedelin standards **b** and samples in CMCs of *T. wilfordii*.

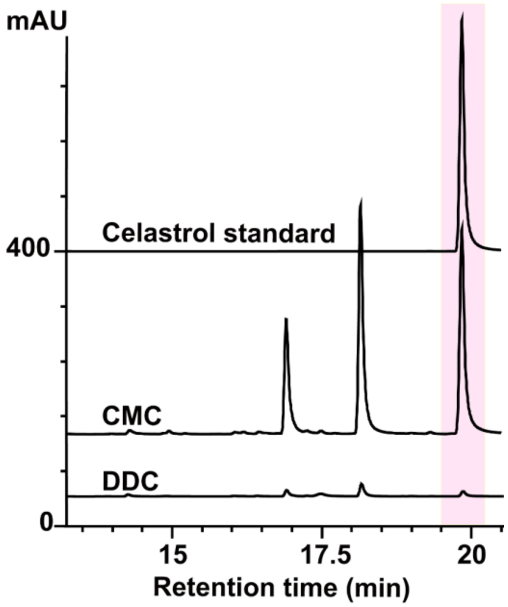

Figure S4 UPLC chromatograms of celastrol standards and samples.

Table S1 Primers used in this study

Usage	Primer Name	Primer Sequence (5'-3')	
Vectors construction	OE-SQS	F-primer	CACCATGGGGAGTTTGTGGACGATG
		R-primer	CTACTTATTTGGCCGGTCGAGGGAC
	OE-SE	F-primer	CACCATGGTGGTGATCGATCGGTAT
		R-primer	TCAATCGACAGGAGGAGCTCGAACT
	OE-OSC	F-primer	CACCATGTGGAAGCTCAAAGTTGCT
		R-primer	TCAATAGCCTTTGGATGGTAACGGA
	M13	F-primer	TGTAAAACGACGGCCAGT
		R-primer	CAGGAAACAGCTATGACC
	pH7	F-primer	TCATTTGGAGAGGACTCCGG
	Hyg	F-primer	ATCAGCTTGCATGCCGGTC
		R-primer	ATCATACATGAGAATTAAGGGAGTCAC
qRT-PCR	Ef1α	F-primer	CCAAGGGTGAAAGCAAGGAGAGC
		R-primer	CACTGGTGGTTTTGAGGCTGGTATCT
	QSQS	F-primer	AATAACACAGCGAATGGG
		R-primer	GGGACTTAGGTATCTCGTTTA
	QSE	F-primer	CGGTCGCATCTACCATAAATACTCTG
		R-primer	CCAATCCACAAGCGTTTAGGC
	QOSC	F-primer	GGTTACCCCCAACAGGAAATC
		R-primer	TGGATGGTAACGGAACACGC
	QDXS	F-primer	GGCGACTACTGGGTCTTTCTT
		R-primer	TGTCTTTGCGTATCATCATCCT
	QHMGS	F-primer	CTGGAGGTAGGGAGCGAGAC
		R-primer	CCATAGCAGGCATTGGTTGA
	QHMGR	F-primer	GCCTTTGCTGCTGGACGACTA

	R-primer	CCGCTGCTCTTCTGGCTGAC
QGGPPS	F-primer	GGCAAGAGGGTTCGTCCAG
	R-primer	AAAGTAGTGCATCACCAGCAAG
QFPS	F-primer	CAGACCCTCACCTTCCATT
	R-primer	AAGAGTAACCATAAGCAGCAGAC
QIDI	F-primer	GTCCCTTCCACCCTAACC
	R-primer	GCCCAACCACACGATCATTC
